# Mitochondrial HMG‐CoA Synthase Deficiency Presenting as Pediatric Metabolic Stroke: A Case Report of a Novel Homozygous HMGCS2 (p.Ile56Asn) Variant

**DOI:** 10.1002/ccr3.71622

**Published:** 2025-12-09

**Authors:** Yasmeen Alshami, Osama Hroub, Mohammad Hroub, Saja Abouodeh, Zahra Makhamre, Ahmad G. Hammouri, Ibrahim Alzatari, Osama Atawneh

**Affiliations:** ^1^ Faculty of Medicine Palestine Polytechnic University Bethlehem Palestine; ^2^ Faculty of Medicine Palestine Polytechnic University Hebron Palestine; ^3^ Radiology Department Al‐Ahli Hospital Hebron Palestine; ^4^ Al‐Ahli Hospital Hebron Palestine; ^5^ PRCS Hospital Hebron Palestine

**Keywords:** dystonia, HMG‐CoA synthase deficiency, metabolic disorders, mitochondrial disease, pediatric neurology

## Abstract

Mitochondrial HMG‐CoA synthase deficiency should be suspected in infants with hypoketotic hypoglycemia, metabolic acidosis, and basal ganglia lesions. A 2‐year‐old boy with a novel HMGCS2 variant presented with refractory seizures and encephalopathy, highlighting the need for rapid metabolic and genetic evaluation for timely management.

## 1 Introduction

Mitochondrial 3‐hydroxy‐3‐methylglutaryl‐CoA synthase deficiency (mHS deficiency) is an ultrarare autosomal recessive ketogenesis disorder caused by pathogenic variants in HMGCS2, the gene encoding the mitochondrial isoform of HMG‐CoA synthase [1]. During fasting, illness, or any catabolic stress, ketone bodies provide a critical alternative fuel for the brain; therefore, impaired ketogenesis results in hypoketotic hypoglycemia, high anion gap metabolic acidosis, encephalopathy, and hepatic dysfunction [1, 2].

Neuroimaging in affected children may reveal characteristic bilateral basal ganglia injury, particularly involving the caudate and putamen, reflecting their metabolic vulnerability during energy failure [3]. Clinically, mHS deficiency is frequently misdiagnosed as infectious or epileptic encephalopathy because presenting symptoms such as seizures, altered mental status, and metabolic acidosis overlap with more common pediatric emergencies [2, 3]. For this reason, the presence of nonketotic hypoglycemia, severe acidosis, and basal ganglia involvement should prompt evaluation for ketogenesis defects. With the increasing accessibility of genetic testing, several novel HMGCS2 variants have been identified, broadening the known phenotypic and genotypic spectrum of this disorder [4].

Early recognition and rapid initiation of metabolic stabilization, particularly high glucose infusion, are essential to prevent neurological injury. We report a previously healthy 2‐year‐old boy who developed acute encephalopathy, refractory seizures, dystonia, profound metabolic acidosis, hyperlipidemia, and bilateral basal ganglia necrosis following a febrile illness. Genetic analysis identified a novel homozygous HMGCS2 missense variant (p.Ile56Asn), consistent with mitochondrial HMG‐CoA synthase deficiency [4]. This case highlights the importance of recognizing biochemical warning signs of impaired ketogenesis and integrating metabolic, radiologic, and genetic findings for timely diagnosis and management.

## 2 Case Presentation

A previously healthy 2‐year‐old boy presented with a 3‐day history of high‐grade fever (up to 39°C axillary) and nonbloody diarrhea, experiencing seven to eight episodes daily. Initially, he was treated at a local healthcare facility for presumed acute gastroenteritis with oral rehydration therapy, antibiotics (cefdinir and metronidazole), and antipyretics. Despite these interventions, his symptoms persisted, and his oral intake significantly declined.

The patient's medical history was unremarkable, with no known neurological or metabolic disorders in the family. The psychosocial background was noncontributory. Given the atypical clinical course and failure to respond to conventional treatment, investigations into possible inborn errors of metabolism were pursued. Genetic analysis subsequently identified a homozygous missense variant in the HMGCS2 gene (c.167 T>A, p.Ile56Asn), initially classified as a variant of uncertain significance.

On the fourth day of illness, the child was found unresponsive with abnormal movements characterized by teeth clenching and frothy oral secretions. Upon arrival at the referring hospital, he was febrile with a Glasgow Coma Scale (GCS) score of 4 out of 15. Ophthalmologic examination revealed slightly pale optic discs with sluggish pupillary reflexes. During hospitalization, he developed persistent generalized dystonia. Electroencephalogram (EEG) demonstrated diffuse encephalopathy with later prominence of slow waves over the left hemisphere. Hemodynamic status was stable except for transient hypotension following intubation, which was managed with dopamine infusion.

Initial neuroimaging with noncontrast computed tomography (CT) of the brain and cerebrospinal fluid (CSF) analysis was unremarkable. Seizures refractory to benzodiazepines and phenytoin prompted transfer to a tertiary care center for specialized management.

Upon admission to our center, the patient remained encephalopathic (GCS 3 to 4 out of 15), necessitating endotracheal intubation and mechanical ventilation. A comprehensive septic workup was conducted, including CSF analysis and blood and urine cultures, all of which were negative. Empiric intravenous antibiotics (ceftriaxone) and antiviral therapy (acyclovir) were initiated. Negative polymerase chain reaction (PCR) testing for herpes simplex virus and enterovirus led to discontinuation of these treatments.

### 2.1 Differential Diagnosis, Investigations, and Treatment

Laboratory investigations revealed severe metabolic derangement. Venous blood gas analysis demonstrated a profoundly high anion gap metabolic acidosis (pH 6.9, bicarbonate 3.4 mmol/L, partial pressure of carbon dioxide 11 mmHg, anion gap 24), accompanied by markedly elevated lactate (16.6 mmol/L). Additional tests showed nonketotic hypoglycemia, severe hypertriglyceridemia (2900 mg/dL), hypercholesterolemia (375 mg/dL), elevated liver enzymes (aspartate aminotransferase 417 U/L, alanine aminotransferase 219 U/L), and increased creatine phosphokinase (590 U/L), indicating multiorgan involvement.

**FIGURE 1:**
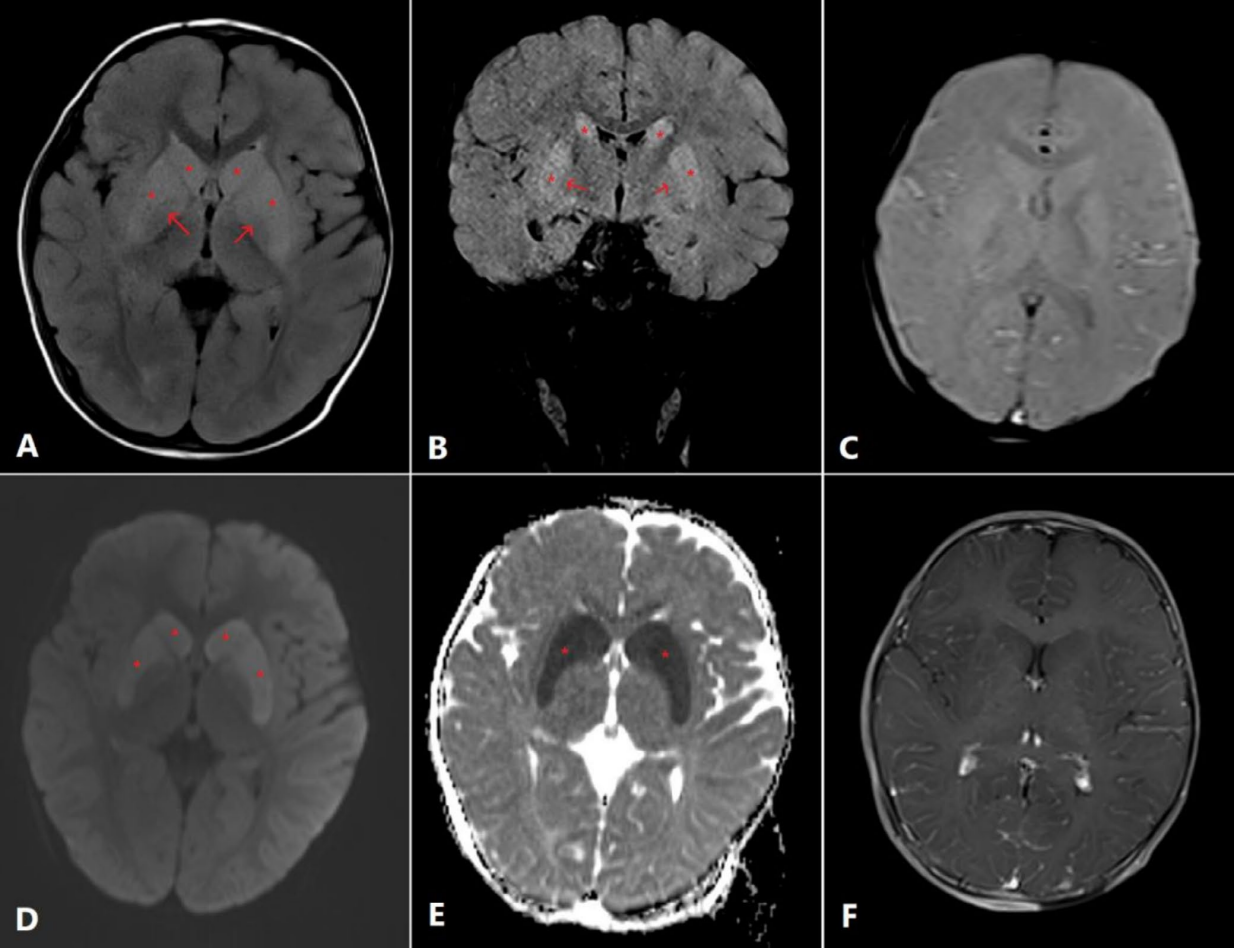
(A, C–F) Selected axial and (B) coronal cuts of the patient's MRI [A: Axial Fluid attenuated inversion recovery (FLAIR) image, B: Coronal FLAIR image, C: Axial Susceptibility weighted imaging (SWI), D: Diffusion‐weighted image (DWI), E: Apparent diffusion coefficient (ADC), F: Axial T1 fat sat/FS with contrast]. There is bilateral symmetrical increased T2/FLAIR signal and edema involving the lentiform and caudate nuclei (asterisks in A and B) with relative sparing of the globus pallidus (solid arrows in A and B) associated with diffusion restriction (high DWI signal—asterisks in D, with corresponding low ADC value—asterisks in E). No associated postcontrast enhancement (F) or blooming artifact on the SWI (C).

Magnetic resonance imaging (MRI) of the brain revealed bilateral symmetrical necrosis of the caudate and putamen nuclei. The affected areas exhibited increased signal intensity on T2‐weighted and fluid‐attenuated inversion recovery (FLAIR) sequences, diffusion restriction on diffusion‐weighted imaging (DWI), and low apparent diffusion coefficient (ADC) values. Notably, there was no evidence of blooming artifact or contrast enhancement. These findings are highly suggestive of metabolic stroke, consistent with mitochondrial dysfunction (Figure 1). This imaging is pivotal for clinicians as it highlights basal ganglia vulnerability in metabolic encephalopathies, aiding differentiation from infectious or ischemic causes and guiding targeted metabolic investigations.

During hospitalization, the patient experienced a secondary febrile episode with elevated C‐reactive protein (193 mg/L). Repeat cultures identified a *Klebsiella* species urinary tract infection, successfully treated with a 14‐day course of piperacillin–tazobactam. The patient remained hemodynamically stable, was extubated after 2 days, and was gradually weaned off supplemental oxygen.

Persistent oropharyngeal dysphagia necessitated surgical insertion of a gastrostomy tube to ensure adequate long‐term enteral nutrition. Management of ongoing dystonia included oral administration of baclofen and trihexyphenidyl via the gastrostomy tube. Seizure prophylaxis was maintained with levetiracetam, reflecting current standards for controlling refractory seizures in metabolic encephalopathies.

Follow‐up electroencephalograms continued to demonstrate encephalopathic features, predominantly slow wave activity over the left hemisphere. Unfortunately, repeat metabolic testing and neuroimaging prior to discharge were unavailable. At discharge, the patient exhibited improved general condition with stabilized vital signs, enhanced consciousness, and tolerance of enteral medications. Biochemical markers, including liver enzymes, lipid profile, and lactate levels normalized; however, generalized dystonia persisted, indicating a guarded long‐term neurological prognosis.

This case underscores the diagnostic complexity of distinguishing metabolic encephalopathy from infectious etiologies such as postinfectious encephalitis. The delay in recognizing an underlying inborn error of metabolism, supported retrospectively by neuroimaging and biochemical abnormalities, emphasizes the necessity for high clinical suspicion in similar pediatric presentations. Identification of the homozygous HMGCS2 missense variant consolidated the diagnosis of mitochondrial 3‐hydroxy‐3‐methylglutaryl‐CoA synthase deficiency, a rare but critical cause of impaired ketogenesis.

## 3 Conclusion and Results (Outcome and Follow‐Up)

This case illustrates the complex diagnostic challenges and severe clinical manifestations associated with mitochondrial 3‐hydroxy‐3‐methylglutaryl‐CoA synthase deficiency. A diagnosis of a ketogenesis disorder should be strongly considered in patients presenting with nonketotic hypoglycemia, high anion gap metabolic acidosis, and bilateral basal ganglia necrosis. Although the HMGCS2 variant (p.Ile56Asn) is currently classified as a variant of uncertain significance due to unconfirmed pathogenicity, its clinical and radiologic features closely resemble those reported in confirmed cases, supporting its probable pathogenic role.

The patient's condition stabilized following prompt metabolic intervention with high‐dose glucose infusion and correction of acid–base imbalance. Nevertheless, the neurological prognosis remains guarded due to persistent dystonia and basal ganglia injury. This case underscores the critical importance of integrating metabolic assessments, genetic testing, and neuroimaging in pediatric patients with unexplained encephalopathy. Moreover, it emphasizes the value of early genetic evaluation in suspected inborn errors of metabolism to facilitate timely diagnosis and guide appropriate management.

## 4 Discussion

Acute metabolic encephalopathies present diagnostic obstacles because patients need prompt empirical treatment alongside complete diagnostic assessment while experiencing rapid health decline. Hypoketotic hypoglycemia (glucose 1.8 mmol/L) and severe acidosis (pH 6.9) with lactate 16.6 mmol/L, together with basal ganglia necrosis on MR images in this patient, indicate HMGCS2 deficiency. Impaired hepatic ketone production rates become evident through biochemical testing, as HMGCS2 functions as the rate‐limiting enzyme that converts acetyl‐CoA into HMG‐CoA. Metabolic crisis arises due to three causes following enzyme deficiency: (i) diminished ketone production, (ii) toxic acyl‐CoA accumulation, and (iii) disruptions in the TCA cycle function [5].

Bilateral basal ganglia necrosis in Fatty acid oxidation defects (FAODs) shows metabolic stroke patterns that correspond to the high metabolic needs and poor collateral circulation of these structures [3]. Additional diagnostic complexity occurs because several inborn errors of metabolism display similar clinical features; therefore, it requires exclusion of three main possibilities, consisting of (i) GLUT1 deficiency (normal CSF lactate results), (ii) Leigh syndrome exhibiting brainstem symptoms, and (iii) methylmalonic acidemia revealing elevated C3 concentrations beyond 5 μmol/L. Results from the CSF analysis (2 cells/μL) combined with a normal CT scan ruled out infectious encephalitis and established toxic‐metabolic encephalopathy as the most likely diagnosis. The patient developed extremely high triglyceride levels (2900 mg/dL) because β‐oxidation defects led to increased VLDL synthesis and breakdown of lipids [2].

This variant of unknown significance (VUS) HMGCS2 c.167 T>A (p.Ile56Asn) is an undocumented homozygous variant that seems to alter a fundamental acetyl‐CoA binding domain. Lack of this variant in human populations, coupled with negative computer modeling results, points to a disease‐causing mutation [6]. The presentation of the phenotype resonates with previously documented cases of HMGCS2 deficiency, thus confirming its pathogenic character. Research on analogous domain mutations has shown their tendency to cause pathogenic effects. The clinical and biochemical presentation, alongside the HMGCS2 variant VUS classification, allows genetic testing to demonstrate pathogenicity [1].

Initial management consisted of using three sequential components: (i) high dextrose infusion rate (10 mg/kg/min) and (ii) bicarbonate therapy for acidemia management, followed by (iii) enteral feeding [3]. The disease carries worrisome long‐term implications for patients due to the potential development of neurological harm and fatal metabolic emergencies. The patient's poor outcome can be attributed to dystonia and basal ganglia damage, but trihexyphenidyl shows promise for symptom improvement [4].

The guarded treatment predictions from this situation demonstrate the serious nature of these metabolic abnormalities. Basal ganglia damage combined with dystonic movements appears as a serious sign throughout the patient's development. The presented case demonstrates why medical teams need urgent metabolic control measures combined with sustained neuroprotective approaches to treat this complex condition successfully.

## Author Contributions

**Yasmeen Alshami:** conceptualization, investigation, methodology, supervision, visualization, writing – review and editing. **Osama Hroub:** data curation, investigation, methodology, resources, supervision, visualization, writing – review and editing. **Mohammad Hroub:** data curation, formal analysis, investigation, project administration, resources, writing – original draft. **Saja Abouodeh:** data curation, methodology, resources, software, validation, writing – original draft. **Zahra Makhamre:** investigation, resources, validation. **Ahmad G. Hammouri:** resources, supervision, visualization. **Ibrahim Alzatari:** software. **Osama Atawneh:** supervision, validation, visualization.

## Funding

The authors have nothing to report.

## Consent

Written informed consent was obtained from the patient's family for the publication of this case report.

## Conflicts of Interest

The authors declare no conflicts of interest.

## Data Availability

The data used to support the findings of this study are included in the article.
